# Effect of Functionally Graded Material (FGM) Interlayer in Metal Additive Manufacturing of Inconel-Stainless Bimetallic Structure by Laser Melting Deposition (LMD) and Wire Arc Additive Manufacturing (WAAM)

**DOI:** 10.3390/ma16020535

**Published:** 2023-01-05

**Authors:** Seong-Won Yoo, Choon-Man Lee, Dong-Hyeon Kim

**Affiliations:** 1 Department of Smart Manufacturing Engineering, School of the Smart Manufacturing Engineering, Changwon National University, Changwon 51140, Republic of Korea; 2 Department of Mechanical Engineering, College of Mechatronics, Changwon National University, Changwon 51140, Republic of Korea; 3 Mechatronics Research Center, Changwon National University, Changwon 51140, Republic of Korea

**Keywords:** metal additive manufacturing, directed energy deposition, bimetallic structure, functionally graded material

## Abstract

Bimetallic structures manufactured by direct deposition have a defect due to the sudden change in the microstructure and properties of dissimilar metals. The laser metal deposition (LMD)-wire arc additive manufacturing (WAAM) process can alleviate the defect between two different materials by depositing the functionally graded material (FGM) layer, such as a thin intermediate layer using LMD and can be used to fabricate bimetallic structures at high deposition rates with relatively low costs using WAAM. In this study, the LMD-WAAM process was performed, and the microstructure of the fabricated bimetallic structure of IN625-SUS304L was investigated. The microstructure of the FGM zone of the LMD-WAAM sample was mainly fine equiaxed dendrite morphologies. In contrast, coarse columnar dendrite morphologies constituted the WAAM zone. The composition of the major alloying elements of the LMD-WAAM sample gradually changed with the height of the deposited layer. The microhardness of the LMD-WAAM sample tended to increase with an increasing Inconel content. In the case of the LMD-WAAM sample, the fracture occurred near the interface between 25% IN625 and 0% IN625; in the WAAM sample, the final fracture occurred in SUS304L near the interface. The tensile strength of the LMD-WAAM samples was inversely proportional to the laser power. The results showed that the LMD-WAAM samples had 8% higher tensile strength than the samples fabricated using only WAAM.

## 1. Introduction

A bimetallic structure is a composite material in which two different metals are combined to obtain the advantages of different properties such as the thermo-physical, mechanical, electrical, optical, and corrosion/oxidation resistance properties of the two materials [1]. The development of these structures offers numerous advantages over a single material and allows for a wide range of applications [2,3,4,5]. Additive manufacturing (AM) can fabricate bimetallic structures entirely using multiple metallic materials, and these can also be fabricated using metallic parts prefabricated by conventional manufacturing techniques as a substrate. However, the sudden change in the microstructure and properties of dissimilar metals causes problems such as lack of fusion, deterioration of mechanical properties due to non-uniform melting and rapid solidification, and micro-segregation of alloy elements [6,7,8].

In order to solve this problem, the AM that is used to fabricate various functionally graded materials (FGMs) is being studied [9]. FGM has a gradual change in chemical composition and microstructure from one side to the other, allowing the reduction in the stress concentrations that appear near sharp interfaces between two different phases [10,11]. FGM is advantageous in applications that require different performances in different locations due to continuous or quasi-continuous gradient changes in physical and chemical properties [12,13]. There are several material combinations that have different thermal and mechanical properties used in FGM. Liu et al. [14] fabricated TiC/Ti FGM using laser metal deposition (LMD) and found that this prevented crack initiation more effectively than using homogeneous composites with a high TiC grain content. Bobbio et al. [15] fabricated FGM composed of Ti6Al4V, and Invar was fabricated using direct energy deposition (DED). In the transition region with 32 layers, the volume fraction of Ti6Al4V decreased by 3 vol%, and the volume fraction of Invar increased by 3 vol%. FeTi and Fe_2_Ti phases and cracks were observed in the transition region. The cause of crack formation was suggested to be residual stress due to the coefficient of thermal expansion (CTE) mismatch of the two materials. Zhang et al. [16] fabricated FGM composed of STS316L with five different wt% Cr/Ni alloys; the columnar dendrites grew epitaxially over several layers of Cr/Ni, and no defects were observed in SUS316L. Carrol et al. [17] fabricated FGM SUS304L/IN625 using LMD. Cracks were observed in the area with 50 vol% IN 625 and 79 vol% SUS304L. Energy-dispersive spectroscopy (EDS) mapping showed that the concentrations of Mo, Nb, and C were high in the vicinity of the crack. These elements were rarely observed except near the cracks, suggesting that the cause of the final macroscopic cracking was the formation of compounds between Mo, Nb, and C. The bimetallic structure of IN625, which has excellent high-temperature strength and corrosion resistance, and SUS304L, which has excellent mechanical properties at room temperature and is relatively low cost, has potential advantages arising from the combination of two different materials. For example, in high-temperature and corrosive environment applications such as light–water reactors, gas turbines, and oil-reformer towers, IN625 is used for components that experience high-temperature and oxidative environments, and SUS304L is used for components exposed to relatively lower temperatures or support. AM technology used to fabricate the IN625-SUS304L bimetal can be divided into two types: powder bed-based additive manufacturing and powder/wire-fed additive manufacturing.

LMD is a method in which powder is fed into a molten pool under a moving laser [18]. LMD can fabricate FGMs using two or more powder feeders to change the volume fraction of different materials or pre-mixed powders to change the interlayer powder composition [19]. WAAM is an additive manufacturing technology that uses arc-based thermal energy and wire-type feedstock [20]. It can process a wide range of metals using various welding wires for deposition and can manufacture large-scale parts with high deposition rates and low raw material and equipment costs [21]. However, bimetal parts manufactured by direct deposition show residual stress and deformation due to differences in the thermal expansion of materials, making it difficult to manufacture with WAAM [22]. These problems can be alleviated with the LMD-WAAM process. By fabricating FGM using LMD on the substrate and then fabricating it using WAAM, the interlayer residual stress between two dissimilar materials can be relieved [23,24,25], and a bimetallic structure can be manufactured at a high deposition rate and low cost.

Most of the studies on the LMD-WAAM mainly focus on WAAM fabrication of medium and large components used as substrates for subsequent deposition and then use LMD to fabricate components with more complex or smaller size features [26,27]. The LMD-WAAM process, in which the FGM layer fabricated by LMD is used as an intermediate layer of the bimetal structure and then the material is deposited on the FGM layer by WAAM, has not been studied in depth. The fabrication of bimetallic structures using LMD-WAAM is slower than that using WAAM alone but has the advantage of residual stress relief. In the experiments, the LMD process time was minimized by depositing an FGM layer as a thin intermediate layer.

Therefore, in this study, we evaluated the feasibility of the LMD-WAAM process. The microstructure, tensile properties at room temperature, and microhardness of the bimetallic structure fabricated by LMD-WAAM were investigated.

## 2. Materials and Methods

### 2.1. Materials Preparation

**Figure 1:**
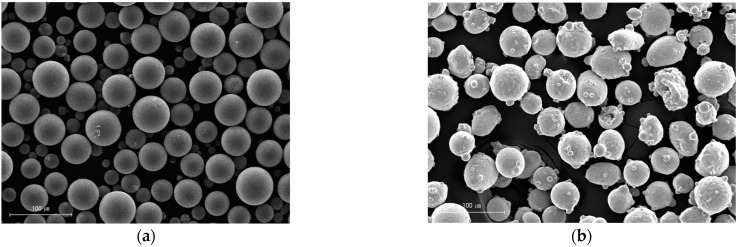
SEM image of the (**a**) Inconel 625 powder and (**b**) 304 L stainless powder.

SUS304L with a size of 30 mm × 60 mm × 50 mm was used as an experimental substrate material. Inconel 625, which is a nickel-based superalloy, and SUS304L spherical powders were used for the LMD process; their composition is shown in Table 1. The morphology and particle size distribution of the two raw material powders are shown in Figure 1. The SUS304L and IN625 powders (particle size 50–150 μm) were initially weighted separately and then mixed with a mechanical stirrer for 20 min according to the desired weight percentage. For the WAAM process, an IN625 wire of 1.2 mm in diameter was used.

### 2.2. Fabricating Samples of Bimetallic Structure Using LMD-WAAM Process

**Figure 2:**
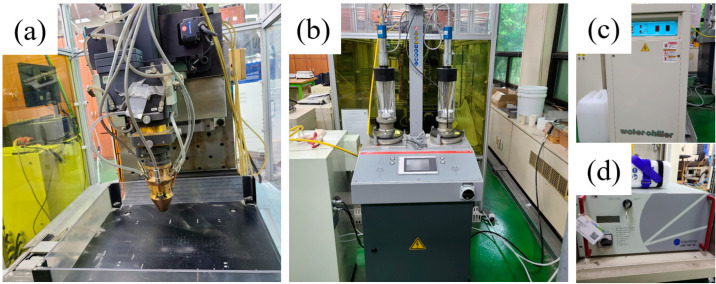
LMD experimental setup: (**a**) AM system; (**b**) powder feeder; (**c**) water chiller; and (**d**) 1 kW diode laser.

**Figure 3:**
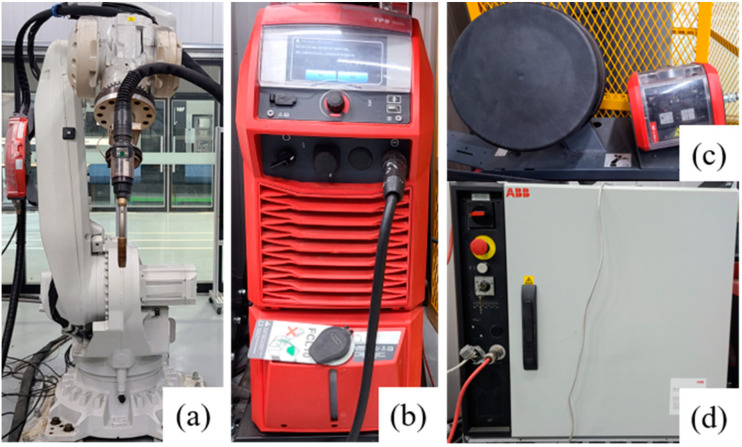
WAAM experimental setup: (**a**) Robot; (**b**) CMT welding machine; and (**c**) wire feed unit; (**d**) controller.

In the LMD process, a machine tool (Laytools, AK390, Burgdorf, Switzerland) equipped with a high-power diode laser deposition head was used. The maximum laser power was 1 kW at a wavelength of 980 nm. The laser focus was calibrated with a lens with a focal length of 198 mm and a laser beam diameter of 3 mm. An additive head with a three-way powder conveying pipe was used to feed the powder to the melt pool, and nitrogen was used as the powder feed gas. Movement in the x and y axes (horizontal plane) was controlled by a computerized numerical control (CNC) table. Argon was supplied as a shielding gas while the powder was being deposited. The LMD experimental setup is shown in Figure 2. The WAAM experiments were conducted using a 6-axis robot arm (ABB Ltd., IRB 6700 of ABB Ltd., Zurich, Switzerland) and a welding machine (TPS 500i, Fronius Co., Ltd., Pettenbach, Austria) for cold metal transfer (CMT) (Figure 3). In the LMD-WAAM experiments, the laser power values were chosen as shown in Table 2 (for LMD) based on previous experience with experimental setups showing good-quality tracks, and an optimal combination of process parameters was used as shown in Table 3 (for WAAM).

**Figure 4:**
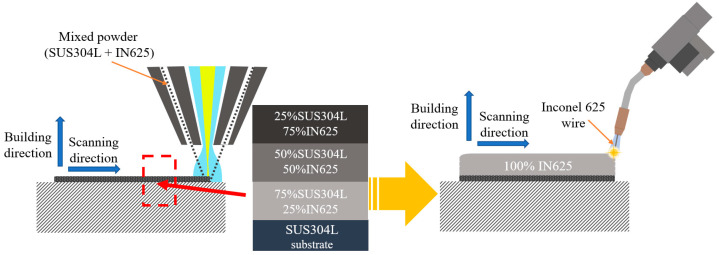
The schematic of the LMD-WAAM process.

**Figure 5:**
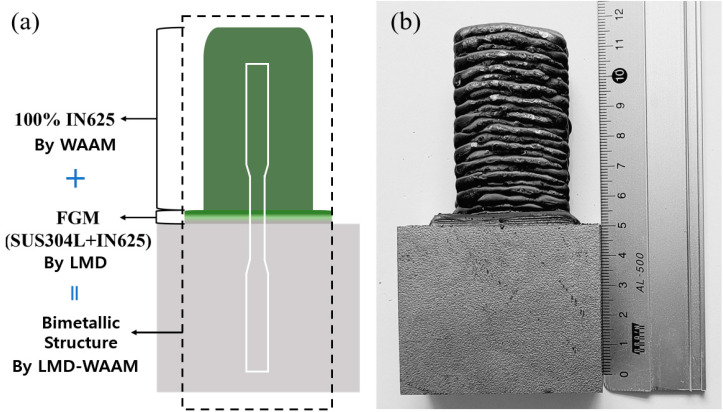
(**a**) The schematic of the LMD-WAAM sample; and (**b**) the photograph of the LMD-WAAM sample (800 W).

Figure 4 shows the workflow of the manufacture of the bimetallic structure using the LMD-WAAM process. First, SUS304L/IN625 FGM with a size of 25 × 45 × 2 mm was deposited on the SUS304L substrate by changing the mixing ratio of 25 wt% per 2 layers using LMD. The mixing ratio was selected to exclude the composition range where defects were found in previous studies [28], and the processing time was minimized by minimizing the number of layers. In the WAAM experiment, 28 layers of Inconel 625 were deposited using the fabricated FGM as a substrate, and Figure 5 shows the LMD-WAAM sample. An interlayer dwell time of 60 s was considered during deposition to prevent the collapse of the material and to prevent the walls from protruding from the end of the layer during welding due to high temperatures. For the tensile properties test, at least three specimens were prepared by machining the manufactured bimetallic structure. The tensile tests were carried out using a universal testing machine (Zwick, Z250 machine, Ulm, Germany) at room temperature. For the metallographic analysis, polishing work was carried out using SiC sandpaper (220 to 2000 grit) after wire electrical discharge machining and etched by Kalling’s No. 2 (5 g CuCl_2_, 100 mL HCl, and 100 mL C2H5OH). An optical microscope (Olympus, Tokyo, Japan) was used for microstructural examination. The images were taken by optical microscope and were converted by a program that was used to calculate the percentage of porous areas of the sample. The compositions were analyzed using an EDS instrument attached to the FE-SEM (MIRA-LMH).

## 3. Results and Discussion

### 3.1. Microstructure

**Figure 6:**
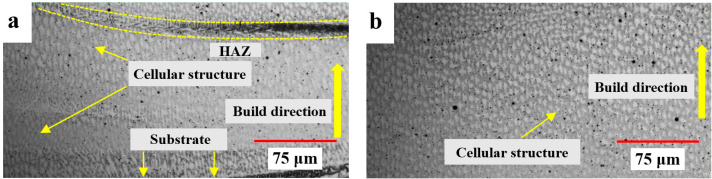

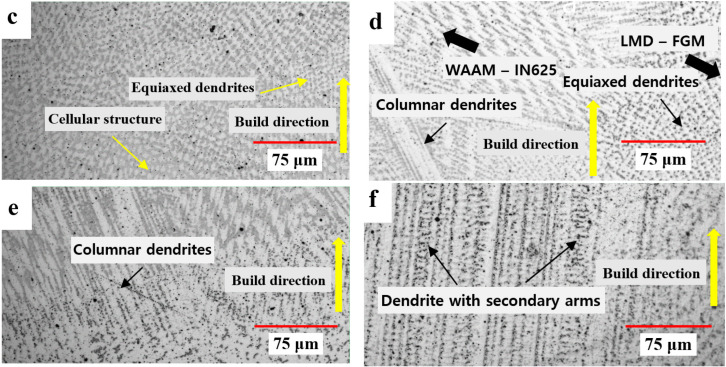
Microstructure of the LMD-WAAM sample (800 W): (**a**) The bottom region of the FGM layer; (**b**) the middle region of the FGM layer; (**c**) the upper region of the FGM layer; (**d**) the bottom region of the WAAM layer; (**e**) the middle region of the WAAM layer; and (**f**) the upper region of the WAAM layer.

In previous studies, too many layers were deposited to fabricate a bimetallic structure with a process using powder materials, resulting in defects or a long process time due to the complicated process. In the case of directly depositing a bimetallic structure using a wire material, defects occurred due to differences in thermal and mechanical properties of different materials. In this study, the advantages of the two processes were utilized by minimizing the LMD process time by depositing the FGM layer as a thin intermediate layer and fabricating the remaining part with WAAM, with a high deposition amount. Figure 6 shows the magnified optical microscopy images of the LMD-WAAM sample fabricated under the condition of 800 W laser power. Some pores were observed meaning this might be due to rapid solidification, which causes gas to be unable to escape [29,30]. The relative densities of the FGM layer and the WAAM layer were measured to be 97.788–98.537% and 99.32–99.44%, respectively. This porosity can be reduced by decreasing the feed rate. there was no evidence of cracks induced by the thermal stresses associated with the LMD and WAAM process as shown Figure 6a. The microstructure of the layer fabricated using WAAM revealed columnar dendrite morphologies. In contrast, the FGM fabricated using LMD showed a structure dominated by equiaxed dendrites morphologies. Figure 6a is the microstructure of the bottom region of the FGM layer, which is the near-substrate region, showing that the cellular structure in most regions was due to high cooling rates. In the upper region (Figure 6c), there were fewer cellular structures than in the middle region (Figure 6b). This could be because the effective surface area for heat dissipation decreased, and the deposition heat of the subsequent layer acted on the previous layer [31]. The FGM layer exhibited a relatively dendrite/cell structure size than that of the WAAM-fabricated layer. It is known that the size of the grain is influenced by the cooling rates during its formation [32]. The finer structure obtained for the LMD-fabricated layer might have been due to higher cooling rates as the gaps in between the unmelted powder particles enhanced heat loss to the environment through radiation and convection [33]. These microstructural features can also be explained through different interrelated energy densities and cooling rates and are related to the different heating powers. The power source for WAAM was much higher than that of LMD, while the scanning speed applied in WAAM was slower, and the ratio of power to scanning speed can be described by the linear energy density (LED) [34].

(1) $$\mathrm{LED}=\frac{P}{V},$$

Here, *P* is the heating power (W) and *V* is the scanning speed (mm/s). This resulted in a much higher heat input in the WAAM process compared to the LMD process. Therefore, when a new Inconel layer was formed with WAAM on the previously deposited FGM layer, heat removal through the preformed layer was slower than that of the LMD due to the accumulated heat, resulting in a relatively coarse structure. Figure 6d shows the microstructure of the layer deposited with WAAM, which was then deposited on the FGM layer, and there were no defects in the bonding interfaces, which indicated that the interface region was not a defective cluster zone. Figure 6e shows the microstructure of the middle region of the WAAM layer and the columnar grain boundaries typical of the WAAM process. Compared with the microstructure of the middle region, the microstructure of the upper region (Figure 6f) shows coarser dendritic crystals, with some secondary dendrites due to continuous accumulation of the heat.

### 3.2. Composition

**Figure 7:**
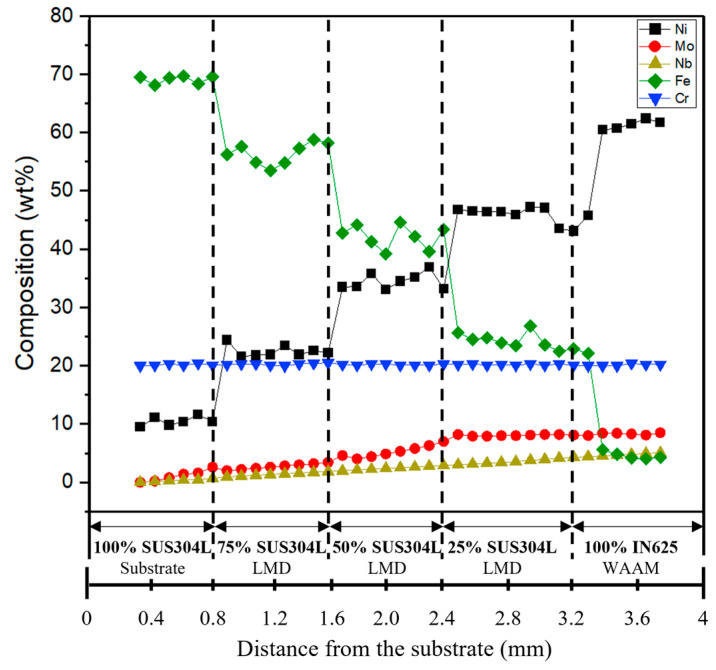
EDS line scanning curves of major element composition along the deposition direction.

Figure 7 shows the composition of the major alloying element results of the FGM (800 W) along the deposition direction measured by EDS; the EDS results demonstrate that the ratios of Fe and Ni elements gradually changed to the intended composition with the height of the deposited layer. Nonetheless, irregular changes appeared near each interface. This might be due to the slight irregularity of the layer thickness of FGM and the difference in the melting temperature between the two materials with different chemical compositions [35].

### 3.3. Tensile Properties

**Figure 8:**
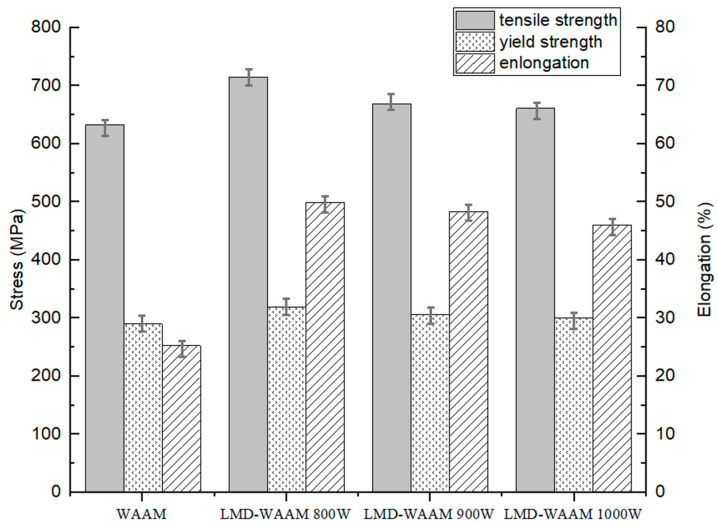
Comparison of UTS, YS, and elongation for WAAM and LMD-WAAM samples (800 W).

**Figure 9:**
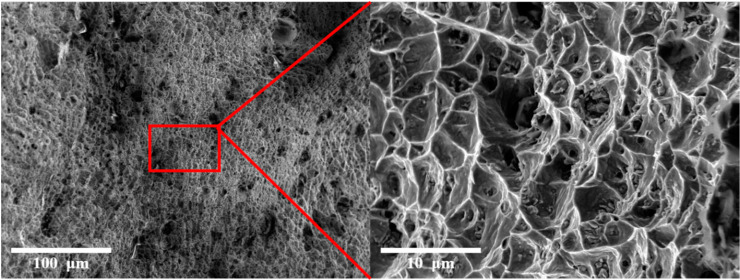
Microscopic appearance of fracture.

Figure 8 shows the average values of YS, UTS, and elongation (%) in the building direction of the bimetallic structure fabricated using WAAM and LMD-WAAM (800 W). It was observed that the tensile strength was improved by the addition of FGM layers at the interface of the bimetallic structure as compared to that without intermediate layers. These results can be attributed to the graded sample in which the stress concentration due to the defect was relieved and the change in chemical composition in the deposited layer at the interface of the two materials. The analysis of the mean values of tensile strength revealed a trend toward a decreasing ultimate tensile strength as the laser power increased. This trend can be explained by the fact that as the heat input increases, the particle size increases and the resistance to the applied load decreases accordingly [36]. In the case of the WAAM sample, the final fracture occurred in SUS304L near the interface, and in the case of the LMD-WAAM sample, the fracture occurred near the interface between 25% IN625 and 0% IN625. This indicates that the interface connection strength between 25% IN625 and 0% IN625 is lower than other interfaces. Representative microscopic appearance of the tensile fracture surface of WAAM and LMD-WAAM fabricated test specimens are shown in Figure 9. The fracture surfaces of all WAAM and LMD-WAAM specimens were characterized by a dimple-like structure showing ductile rupture, and crack growth and propagation due to stress concentration due to internal cracks and pores affecting the fracture mechanism.

### 3.4. Microhardness

**Figure 10:**
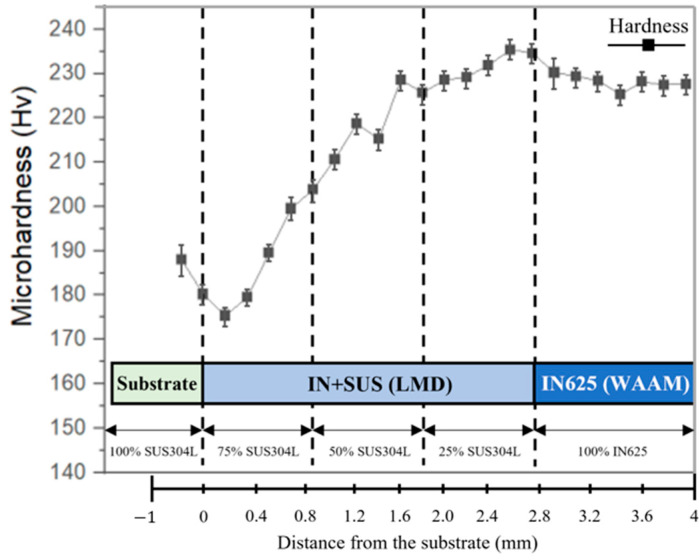
Microhardness distribution along the deposition direction.

The microhardness results are shown in Figure 10. For the LMD-WAAM samples (800 W), the hardness change was smoother as its composition was changed from the FGM layer by the manufactured LMD, unlike the samples deposited by WAAM only. The hardness of the LMD-WAAM sample tended to increase gradually as the mixing ratio of IN625 increased; the contents of Ni, Cr, and Mo increased; the employment strengthening effect for Austenite increased; and the microhardness value increased. The largest microhardness values were observed at 75% IN625 and 100% IN625. However, the microhardness decreased in the region between 25% IN625 and 0% IN625, which might have been caused by Nb segregation and consequential Laves phase formation [37]. Carroll et al. [17] observed a slight decrease in hardness with the introduction of IN625, similar to that observed in this study. This trend in the FGM layer can be seen in the microstructure of the cell structure as shown in Figure 6a. There was also a small decrease in the region deposited by WAAM. This might have been reduced because the microstructure dimension of the WAAM layer was larger than that of the FGM [38].

## 4. Conclusions

In this study, the SUS304L/IN625 bimetallic structure fabricated using the LMD-WAAM process was investigated. Fabricating a bimetal structure using the LMD-WAAM process can have a higher deposition amount than manufacturing only with the LMD process and can have the advantage of alleviating defects compared to manufacturing only with the WAAM process. It was found that LMD-WAAM samples with good mechanical properties and with no evidence of cracks induced by the thermal stresses can be fabricated. The following conclusions can be drawn:

1. The 304 L stainless–Inconel 625 bimetallic structure sample formed by LMD-WAAM fabrication consisted of an FGM zone (304 L stainless-Inconel 625) fabricated using LMD and a WAAM zone (Inconel 625); there was good bonding at the interface with no defects.
2. The microstructure of the FGM zone of the LMD-WAAM sample was mainly fine equiaxed dendrite morphologies. In contrast, the coarse columnar dendrite morphologies were constituted in the WAAM zone, and the upper region showed coarser dendritic crystals with some secondary dendrites.
3. The fracture surfaces of all the samples were characterized by a dimple-like structure showing ductile ruptures. The LMD-WAAM samples had 8% higher tensile strength than the samples fabricated using only WAAM. The LMD-WAAM samples indicated the tendency of a lower laser power to result in higher tensile strength.
4. The ratios of Fe, Ni, and the major alloying elements of the LMD-WAAM sample gradually changed with the height of the deposited layer. The microhardness value of the LMD-WAAM samples decreased slightly in the region between 25% IN625 and 0% IN625, increased with the increasing Inconel content, and then slightly decreased again in the WAAM zone.
5. Finally, Optimizing the LMD-WAAM process can contribute to the improvement of the fabricating a bimetallic structure with high quality.

## Figures and Tables

**Table 1:** Chemical compositions of Inconel 625 and 304L stainless powders [wt. %].

Material	C	Mn	Si	Cr	Mo	Co	Ti	Al	Fe	Ni	Nb
Inconel 625	<0.01	0.36	0.36	21	8.7	0.3	0.07	0.09	<0.1	65.7	3.4
304L stainless	0.017	1.31	0.66	19.5	-	-	-	-	68.9	9.4	-

**Table 2:** Deposition parameters of laser metal deposition.

Laser Power (W)	Scanning Speed (mm/s)	Powder Feed Rate (g/min)	Argon Gas Flow (L/min)	Nitrogen Gas Flow (L/min)
800, 900, 1000	14	8	25	5

**Table 3:** Deposition parameters of wire arc additive manufacturing.

Wire Feed Speed (m/min)	Welding Speed (mm/s)	Average Welding Current (A)	Average Welding Voltage (V)	Dwell Time (s)
4.5	7	161	14	60

## Data Availability

Data presented in this article are available upon request from the corresponding author.
